# Clinical and Demographic Characteristics of Hospitalized Patients With Bronchial Asthma at Rabia Balkhi Hospital in Kabul, Afghanistan: A Retrospective Observational Study

**DOI:** 10.7759/cureus.111098

**Published:** 2026-06-18

**Authors:** Neiloofar Mohmand, Abdul Kafi Muzafar, Zahida Roain, Lailoma Habibi Oria

**Affiliations:** 1 Internal Medicine, Kabul Medical University, Kabul, AFG; 2 Internal Medicine, Rabia Balkhi Hospital, Kabul, AFG

**Keywords:** asthma exacerbation, bronchial asthma, epidemiology, hospitalization, respiratory symptoms

## Abstract

Background

Asthma is a chronic inflammatory airway disease characterized by reversible airflow obstruction and airway hyperresponsiveness. It contributes significantly to global morbidity and hospital admissions, particularly in low-resource settings. This study aimed to describe the clinical and demographic characteristics of patients hospitalized with bronchial asthma at Rabia Balkhi Hospital in Kabul, Afghanistan.

Methods

This retrospective observational study was conducted at Rabia Balkhi Hospital in Kabul, Afghanistan. Medical records of patients admitted during 1397 (March 2018 to March 2019) were reviewed. Thirty-six patients with bronchial asthma were included. Data regarding demographics, residence, occupation, seasonal distribution, and clinical features were analyzed using descriptive statistics.

Results

Among 1,393 hospital admissions, 36 patients (2.58%) were hospitalized with asthma. All hospitalized patients were adult females, 36 (100%). The most affected age group was 25-34 years, accounting for 11 (30.5%) cases. Most patients were residents of Kabul, accounting for 20 (55.5%) cases, while 28 (77.7%) were housewives. Admissions increased during the autumn months. The most common symptom was shortness of breath in 36 (100%) patients, followed by cough in 31 (86.1%), sputum production in 28 (77.7%), anxiety in 25 (69.4%), and fever in 22 (61.1%) patients.

Conclusions

Asthma accounted for a notable proportion of hospital admissions and predominantly affected young adult women. Seasonal variation was observed among hospitalized patients. Environmental exposures, healthcare access, health literacy, and medication adherence may influence asthma outcomes; however, these factors were not directly measured in the present study and warrant further investigation.

## Introduction

Asthma is a chronic inflammatory disease of the airways characterized by reversible airflow obstruction, airway hyperresponsiveness, and recurrent respiratory symptoms such as wheezing, cough, chest tightness, and shortness of breath [1]. It is one of the most common chronic respiratory diseases worldwide and contributes significantly to morbidity, hospital admissions, and healthcare burden, particularly in low- and middle-income countries [2,3].

Asthma exacerbations are influenced by multiple factors, including respiratory infections, environmental pollution, occupational exposure, seasonal variation, smoking, poor medication adherence, and limited access to healthcare services [4,5]. In developing countries, additional challenges such as low health literacy, delayed diagnosis, and inadequate asthma control contribute to increased hospitalization and disease complications [6].

Despite the growing burden of respiratory diseases in Afghanistan, limited published data are available regarding the demographic and clinical characteristics of hospitalized asthma patients [7]. Understanding the patterns of asthma-related admissions may help improve disease management strategies, patient education, and healthcare planning [8].

This retrospective observational study aimed to evaluate the clinical and demographic characteristics of adult patients hospitalized with bronchial asthma at Rabia Balkhi Hospital in Kabul, Afghanistan, during a one-year period.

## Materials and methods

Study design and setting

This retrospective observational study was conducted at Rabia Balkhi Hospital, a tertiary care hospital in Kabul, Afghanistan. The study evaluated the demographic and clinical characteristics of patients hospitalized with bronchial asthma during the year 1397 (March 2018 to March 2019). Rabia Balkhi Hospital primarily provides healthcare services to women; therefore, the study population consisted exclusively of female patients and should not be interpreted as representing the overall distribution of asthma among the general Afghan population.

Study population

Medical records of patients admitted to the Internal Medicine Department with a diagnosis of bronchial asthma were reviewed. Patients with incomplete medical records or uncertain diagnoses were excluded from the study. The diagnosis of bronchial asthma was based on the treating physician's documented diagnosis in the medical records, supported by the clinical history and examination findings recorded at the time of admission.

Data collection

Data were collected retrospectively from hospital medical records using a structured data collection form. The following variables were reviewed and analyzed: age, sex, residence, occupation, seasonal distribution of admissions, and clinical manifestations and presenting symptoms.

Statistical analysis

Data were analyzed using descriptive statistics. Frequencies and percentages were calculated for categorical variables, and results were presented as N (%) in tables and text where appropriate.

Ethical considerations

Ethical approval for this retrospective observational study was waived by the administration of Adei Plus Hospital due to the use of anonymized patient medical records and the low-risk nature of the study. No direct patient interaction or intervention occurred, and patient confidentiality was maintained throughout the study.

## Results

During the study period, a total of 1,393 patients were admitted to the Internal Medicine Department of Rabia Balkhi Hospital. Among these admissions, 36 patients (2.58%) were hospitalized with a diagnosis of bronchial asthma.

The demographic characteristics of hospitalized asthma patients are summarized in Table 1. All hospitalized patients were adult females, accounting for 36 (100%) cases. The most affected age group was 25-34 years, accounting for 11 (30.5%) cases. Most patients were residents of Kabul province, accounting for 20 (55.5%) cases, while 28 (77.7%) were housewives.

**Table 1:** Demographic characteristics of patients hospitalized with bronchial asthma

Variable	Frequency (n)	Percentage (%)
Total asthma patients	36	100
Female patients	36	100
Age group 25-34 years	11	30.5
Residents of Kabul	20	55.5
Housewives	28	77.7

Seasonal variation in hospital admissions was observed, with the highest frequency of asthma-related admissions occurring during the autumn season, as shown in Table 2.

**Table 2:** Seasonal distribution of asthma admissions

Season	Frequency n (%)
Autumn	14 (38.9%)
Spring	8 (22.2%)
Summer	7 (19.4%)
Winter	7 (19.4%)

The clinical manifestations of hospitalized asthma patients are summarized in Table 3. Shortness of breath was the most common presenting symptom, reported in all 36 (100%) patients. Other common symptoms included cough in 31 (86.1%) patients, sputum production in 28 (77.7%), anxiety in 25 (69.4%), and fever in 22 (61.1%) patients.

**Table 3:** Clinical manifestations of patients hospitalized with bronchial asthma

Symptom	Frequency n (%)
Shortness of breath	36 (100%)
Cough	31 (86.1%)
Sputum production	28 (77.7%)
Anxiety	25 (69.4%)
Fever	22 (61.1%)

The findings demonstrate that bronchial asthma represented a notable cause of hospitalization among adult female patients in this setting, with respiratory symptoms being the predominant clinical presentation.

## Discussion

This retrospective observational study evaluated the demographic and clinical characteristics of adult patients hospitalized with bronchial asthma at Rabia Balkhi Hospital in Kabul, Afghanistan. The findings demonstrated that asthma represented a notable cause of hospitalization among patients admitted to the Internal Medicine Department and predominantly affected young adult female patients.

In the present study, all hospitalized asthma patients were female, and the most affected age group was 25-34 years. It is important to note that Rabia Balkhi Hospital primarily provides healthcare services to women; therefore, the exclusively female study population reflects the hospital's patient population and should not be interpreted as representing the overall distribution of asthma in the general Afghan population. Similar observations regarding increased asthma-related healthcare utilization among adult women have been reported in previous epidemiological studies conducted in developing countries [2,3]. Hormonal influences, indoor air pollution, household smoke exposure, and differences in healthcare-seeking behavior may contribute to this pattern [5,8].

Shortness of breath was the most common presenting symptom, followed by cough and sputum production. These findings are consistent with the established clinical manifestations of bronchial asthma reported in previous studies [1,5,7]. The predominance of severe respiratory symptoms among hospitalized patients may reflect delayed presentation to healthcare facilities or inadequate long-term disease control. Similar associations between poor asthma control and increased hospitalization have been reported in regional and international studies [4,6].

The observed increase in asthma admissions during autumn may reflect the influence of seasonal environmental factors, respiratory tract infections, allergen exposure, and air pollution. Seasonal variation in asthma exacerbations has been documented in several epidemiological studies and is commonly associated with climatic changes, airborne allergens, and increased respiratory infections [2,4]. Environmental exposures may therefore play a role in asthma exacerbations requiring hospitalization.

Although these factors were not directly measured in the present study, previous research suggests that limited healthcare access, low health literacy, financial barriers, and inadequate patient education may contribute to poor asthma control in low-resource settings [5,8]. Many patients use medications only during symptomatic periods and may not fully understand the preventive role of maintenance therapy. These factors may contribute to recurrent exacerbations, emergency admissions, and increased healthcare burden.

This study has several limitations. The sample size was relatively small, and the study was conducted at a single center, which may limit the generalizability of the findings. In addition, the retrospective design depended on the accuracy and completeness of medical records. The diagnosis of asthma was based on documentation in patient records, and objective pulmonary function measurements were not consistently available. Furthermore, symptoms such as anxiety and fever were recorded from clinical documentation and were not assessed using standardized measurement tools. The absence of long-term follow-up data also limited further assessment of disease severity and treatment outcomes.

Despite these limitations, this study provides important baseline clinical and demographic data regarding hospitalized asthma patients in Afghanistan, where published respiratory epidemiological data remain limited. The findings emphasize the importance of improving asthma awareness, early diagnosis, and access to appropriate management strategies. Further multicenter studies with larger sample sizes are recommended to better characterize the burden and clinical patterns of asthma in the Afghan population.

## Conclusions

Bronchial asthma represented a notable cause of hospitalization among patients admitted to the Internal Medicine Department of Rabia Balkhi Hospital. The disease predominantly affected young adult female patients, and shortness of breath was the most common presenting symptom. Seasonal variation was observed among hospitalized patients. Environmental exposures, healthcare access, health literacy, and medication adherence may influence asthma outcomes; however, these factors were not directly measured in the present study and warrant further investigation.

Improving patient education, early diagnosis, and access to appropriate asthma management strategies may help reduce asthma-related morbidity and hospitalization. Further multicenter studies are recommended to better evaluate the epidemiological and clinical characteristics of asthma in Afghanistan.
